# Comparison of Health Outcomes Among Patients Admitted on Busy vs Less Busy Days for Hospitalists

**DOI:** 10.1001/jamanetworkopen.2021.44261

**Published:** 2022-01-20

**Authors:** Jennifer P. Stevens, Laura A. Hatfield, David J. Nyweide, Bruce Landon

**Affiliations:** 1 Center for Healthcare Delivery Science, Beth Israel Deaconess Medical Center, Boston, Massachusetts; 2 Division for Pulmonary, Critical Care, and Sleep Medicine, Department of Medicine, Beth Israel Deaconess Medical Center, Boston, Massachusetts; 3 Department of Health Care Policy, Harvard Medical School, Boston, Massachusetts; 4 Center for Medicare & Medicaid Innovation, Centers for Medicare & Medicaid Services, Baltimore, Maryland; 5 Division of General Medicine, Department of Medicine, Beth Israel Deaconess Medical Center, Boston, Massachusetts

## Abstract

This cohort study uses Medicare claims data to analyze health outcomes of Medicare patients admitted to the hospital and being treated by hospitalists on busy vs less busy days.

## Introduction

Increasingly, hospitalized patients are cared for by hospitalists.^1^ When caseloads are higher or patients require more acute care than usual, hospitalists may respond to their cognitive and time constraints by shifting diagnostic or procedural work to specialist colleagues, thereby delaying discharges^2^ or missing preventable safety events.^3^ We hypothesized that hospitalists who admit patients on relatively busy days compensate by increasing their use of inpatient resources, such as specialist care, and put off less urgent tasks (thus extending lengths of stay), but that these changes are not associated with patient health outcomes.

## Methods

In this cohort study, we used Medicare claims data to identify hospitalizations in 2018. For these hospitalizations, patient demographic data were obtained from the Chronic Conditions Data Warehouse, which categorizes race and ethnicity as American Indian or Alaska Native, Asian or Pacific Islander, Black or African American, Hispanic, and non-Hispanic White. We restricted admissions to large (≥250 beds) nonfederal hospitals. Hospitalists were defined using established methods.^1^ Each hospitalist must have had at least 8 admissions at a particular hospital during the year to measure his or her busyness. We counted the number of patients for whom a hospitalist provided any Part B evaluation and management (E&M) service on each working day to obtain the hospitalist’s distribution of busyness, weighted by the relative value unit of each E&M visit. The attending hospitalist for a given admission billed the initial E&M visit. We exploited the near-random assignment of hospitalized patients to an attending hospitalist as a source of exogenous variation in patients’ exposure to hospitalist busyness, including by day of the week.^4^ This study was deemed exempt and the need for patient informed consent was by the institutional review board at the Beth Israel Deaconess Medical Center, Boston, Massachusetts, because this study represents secondary use of publically available data. Our study followed the Strengthening the Reporting of Observational Studies in Epidemiology (STROBE) reporting guideline for cohort studies.

We analyzed admissions among the 25 most prevalent medical diagnosis related groups for patients with full fee-for-service coverage who were 66 years or older (eAppendix in the Supplement). Admissions to hospitalists on their busiest admission days (the hospitalist’s busiest 25% of days, averaging ≥6 additional patients) were compared with admissions on less busy days (the other 75%) with respect to inpatient resource use–specialist consultations, total Part B (physician) spending, and length of stay as well as outcomes in the form of discharge to home, all-cause readmission at 7 and 30 days, and all-cause mortality at 30 days from admission. Tests of difference were conducted with *t* tests for continuous variables and χ^2^ tests for categorical variables; *P* ≤ .05 was considered statistically significant and all tests were 2 tailed. Statistical analyses were performed using SAS Enterprise Guide; version 7.15 (SAS Institute Inc).

## Results

We studied 754 160 admissions admitted by 19 428 hospitalists at 959 hospitals. The mean (SD) age of the admitted patients was 80 (8.5) years, and 417 383 (55.3%) were women. With respect to race and ethnicity, 1.6% were Asian, 10.9% were Black, 1.7% were Hispanic, 83.2% wre non-Hispanic white, and 2.5% were listed as Other (which included American Indian, Native American, other, or unknown).

Of 210 743 patients admitted to hospitalists on one of their busiest days, none experienced any meaningful differences in outcomes (Table); however, Medicare beneficiaries admitted on hospitalists’ busiest days had slightly lower resource use (eg, a mean of 1.12 [95% CI, 1.11-1.12] vs 1.13 [95% CI, 1.13-1.13] consultations during the full stay; *P* < .001) that was balanced by longer length of stay (mean of 5.72 [95% CI, 5.70-5.74] vs 5.63 [95% CI, 5.62-5.64] days; *P* < .001), with no material differences in discharge to home (41.1% vs 41.6% of patients; *P* < .001), readmission (eg, for readmission at 30 days, 17.6% of patients admitted on the busiest days vs 17.5% admitted on less busy days; *P* = .31), or mortality rates (10.5% vs 10.7% of patients; *P* < .001).

**Table.** Patient Characteristics, Resource Use, and Outcomes by Quartile of Hospitalist Busyness^a^

Characteristics	No. (%)			Test of difference
	All (N = 754 160)	Busiest admission days (Q4) (n = 210 743)	Less busy admission days (Q1-Q3) (n = 543 417)	
**Patient characteristics**				
Age, mean (SD), y	80.0 (8.5)	80.1 (8.5)	80.0 (8.5)	<.001
Sex				
Female	417 383 (55.3)	117 044 (55.5)	300 339 (55.3)	.03
Male	336 777 (44.7)	93 699 (44.5)	243 078 (44.7)	.03
Race and ethnicity				
Asian	12 378 (1.6)	3410 (1.6)	8968 (1.7)	.32
Black	82 309 (10.9)	22 809 (10.8)	59 500 (11.0)	.12
Hispanic	13 001 (1.7)	3625 (1.7)	9376 (1.7)	.87
Non-Hispanic White	627 604 (83.2)	175 721 (83.4)	451 883 (83.2)	.02
Other race^b^	18 868 (2.5)	5178 (2.5)	13 690 (2.5)	.12
Disabled				
Yes	128 211 (17.0)	35 709 (16.9)	92 502 (17.0)	.42
No	625 949 (83.0)	175 034 (83.1)	450 915 (83.0)	.42
Medicaid dual-eligible				
Yes	186 103 (24.7)	51 881 (24.6)	134 222 (24.7)	.46
No	568 057 (75.3)	158 862 (75.4)	409 195 (75.3)	.46
**Inpatient resources**				
Physician spending, mean (95% CI), $				
First 2 d	605 (604-606)	589 (587-592)	611 (610-613)	<.001
Full stay	1236 (1233-1238)	1219 (1214-1224)	1242 (1239-1245)	<.001
Consultations, mean (95% CI), No.				
First 2 d	0.60 (0.60-0.60)	0.60 (0.59-0.60)	0.60 (0.60-0.61)	<.001
Full stay	1.13 (1.12-1.13)	1.12 (1.11-1.12)	1.13 (1.13-1.13)	<.001
Length of stay, d	5.65 (5.65-5.66)	5.72 (5.70-5.74)	5.63 (5.62-5.64)	<.001
**Outcomes**				
Discharge to home^c^	312 336 (41.4)	86 543 (41.1)	225 793 (41.6)	<.001
Readmission^d^				
7 d	36 092 (6.0)	10 314 (6.0)	25 778 (5.9)	.22
30 d	106 021 (17.5)	30 136 (17.6)	75 885 (17.5)	.31
30-d Mortality^e^	80 288 (10.7)	22 080 (10.5)	58 208 (10.7)	<.001

^a^ Data are based on Medicare claims from 2018 for common medical admissions to large hospitals for fee-for-service Medicare patients at least 66 years of age. Hospitalists were attributed to an admission according to initial evaluation and management billing codes. The busiest admission days were defined as the hospitalist’s busiest 25% of days, averaging 6 or more additional patients (Q4); less busy days were defined as the other 75% of days (Q1-Q3).

^b^ Other race includes American Indian, Native American, other, and unknown.

^c^ Discharge to home excluded stays with inpatient mortality.

^d^ Readmission was measured relative to the discharge date of a patient’s index admission and excluded stays with inpatient mortality.

^e^ Mortality was measured for all admissions from the admission date.

## Discussion

In this large, national cohort study with ample power to detect meaningful differences, we found small differences in hospitalists’ resource use and no substantive differences in patients’ outcomes during their busiest admission days compared with all other admission days for common medical admissions. Prior studies have noted increased risk of harm to patients admitted to full intensive care units or obstetric floors.^5,6^ Our analysis was at the clinician level and compared physicians with their own prior experience, rather than at the medical floor or team level, which may obscure consequences to the patient when other aspects of the care delivery team exceed a maximum number of patients. However, our findings are reassuring that hospitalists provide similar care regardless of their caseload, potentially from additional supportive coverage models or efforts by individual clinicians to offset busy work environments.

Our study has several limitations. First, we used administrative data, which lacks the nuance to discern subtle variations in care and the acuity level of patients. Second, we assigned each admission to an attending hospitalist based on billing practices rather than through Part A claims. Third, we measured busyness for fee-for-service Medicare patients only, which does not capture all of the patients cared for by hospitalists.

In conclusion, the findings of this study indicate that patients admitted to hospitalists on their busiest days received similar care and had outcomes similar to those admitted on less busy days.

## Notes

Supplement. **eAppendix.** Most Common 25 Medical Diagnosis Related Groups
