# Supplementary material for: Comparison of Health Outcomes Among Patients Admitted on Busy vs Less Busy Days for Hospitalists
Source: JAMA Netw Open. 2022 Jan 20;5(1):e2144261. doi: 10.1001/jamanetworkopen.2021.44261 (PMC8777570; doi:10.1001/jamanetworkopen.2021.44261)
Supplement: Supplement. — eAppendix. Most Common 25 Medical Diagnosis Related Groups [file jamanetwopen-e2144261-s001.pdf]

Supplemental Online Content

Stevens JP, Hatfield LA, Nyweide DJ, Landon B. Comparison of health outcomes among patients admitted on busy vs less busy days for hospitalists. *JAMA Netw Open*. 2022;5(1):e2144261. doi:10.1001/jamanetworkopen.2021.44261

eAppendix. Most Common 25 Medical Diagnosis Related Groups

This supplemental material has been provided by the authors to give readers additional information about their work.

© 2022 Stevens JP et al. *JAMA Network Open*.

Appendix 1. Most Common 25 Medical Diagnosis Related Groups

064	INTRACRANIAL HEMORRHAGE OR CEREBRAL INFARCTION W MCC
065	INTRACRANIAL HEMORRHAGE OR CEREBRAL INFARCTION W CC
066	INTRACRANIAL HEMORRHAGE OR CEREBRAL INFARCTION W/O CC/MCC
177	RESPIRATORY INFECTIONS & INFLAMMATIONS W MCC
178	RESPIRATORY INFECTIONS & INFLAMMATIONS W CC
179	RESPIRATORY INFECTIONS & INFLAMMATIONS W/O CC/MCC
190	CHRONIC OBSTRUCTIVE PULMONARY DISEASE W MCC
191	CHRONIC OBSTRUCTIVE PULMONARY DISEASE W CC
192	CHRONIC OBSTRUCTIVE PULMONARY DISEASE W/O CC/MCC
193	SIMPLE PNEUMONIA & PLEURISY W MCC
194	SIMPLE PNEUMONIA & PLEURISY W CC
195	SIMPLE PNEUMONIA & PLEURISY W/O CC/MCC
246	PERC CARDIOVASC PROC W DRUG-ELUTING STENT W MCC OR 4+ VESSELS/STENTS
247	PERC CARDIOVASC PROC W DRUG-ELUTING STENT W/O MCC
280	ACUTE MYOCARDIAL INFARCTION, DISCHARGED ALIVE W MCC
281	ACUTE MYOCARDIAL INFARCTION, DISCHARGED ALIVE W CC
282	ACUTE MYOCARDIAL INFARCTION, DISCHARGED ALIVE W/O CC/MCC
291	HEART FAILURE & SHOCK W MCC
292	HEART FAILURE & SHOCK W CC
293	HEART FAILURE & SHOCK W/O CC/MCC
308	CARDIAC ARRHYTHMIA & CONDUCTION DISORDERS W MCC
309	CARDIAC ARRHYTHMIA & CONDUCTION DISORDERS W CC
310	CARDIAC ARRHYTHMIA & CONDUCTION DISORDERS W/O CC/MCC
377	G.I. HEMORRHAGE W MCC
378	G.I. HEMORRHAGE W CC
379	G.I. HEMORRHAGE W/O CC/MCC
391	ESOPHAGITIS, GASTROENT & MISC DIGEST DISORDERS W MCC
392	ESOPHAGITIS, GASTROENT & MISC DIGEST DISORDERS W/O MCC
602	CELLULITIS W MCC
603	CELLULITIS W/O MCC
640	NUTRITIONAL & MISC METABOLIC DISORDERS W MCC
641	NUTRITIONAL & MISC METABOLIC DISORDERS W/O MCC
682	RENAL FAILURE W MCC
683	RENAL FAILURE W CC
684	RENAL FAILURE W/O CC/MCC
689	KIDNEY & URINARY TRACT INFECTIONS W MCC
690	KIDNEY & URINARY TRACT INFECTIONS W/O MCC
698	OTHER KIDNEY & URINARY TRACT DIAGNOSES W MCC

© 2022 Stevens JP et al. *JAMA Network Open*.

699	OTHER KIDNEY & URINARY TRACT DIAGNOSES W CC
700	OTHER KIDNEY & URINARY TRACT DIAGNOSES W/O CC/MCC
870	SEPTICEMIA OR SEVERE SEPSIS WITH MV >96 HOURS
871	SEPTICEMIA OR SEVERE SEPSIS WITHOUT MV >96 HOURS WITH MCC
872	SEPTICEMIA OR SEVERE SEPSIS WITHOUT MV >96 HOURS WITHOUT MCC

© 2022 Stevens JP et al. *JAMA Network Open*.
